# Intraoperative management of hypoxemia with recruitment maneuvers: are the benefits worth the costs?

**DOI:** 10.1186/cc12045

**Published:** 2013-03-19

**Authors:** V Grosomanidis, B Fyntanidou, K Karakoulas, K Kotzampasi, E Oloktsidou, C Nouris, C Skourtis

**Affiliations:** 1 Aristotle Medical School, Thessaloniki, Greece

## Introduction

Several strategies can be applied for the prevention and management of anesthesia-related hypoxemia. The aim of our study was to investigate the cardiovascular effects of a specific recruitment maneuver (RM) used for the management of intraoperative hypoxemia.

## Methods

Forty-nine patients (29 male/20 female) of mean age 57.4 ± 9.9 years, mean weight 80.2 ± 13.6 kg and ASA-PS classification I to IV (I: 2, II: 27, III: 16, IV: 6), undergoing general surgery procedures, who developed intraoperative hypoxemia (PaO_2_/FiO_2 _<200), were enrolled in our study. For the management of hypoxemia a total of 67 RMs have been applied, which consisted of a manual increase of the airway pressure to 40 cmH_2_O. This rise has been maintained for 10 seconds and was followed by an increase of positive end-expiratory pressure from 5 to 10 cmH_2_O. Before RM application an oesophageal Doppler monitor probe was inserted into the patients for measuring stroke volume (SV), cardiac output (CO), peak velocity (PV) and flow corrected time (FTc). Standard monitoring also included ECG, IBP, ETCO_2 _and SpO_2_. Heart rate (HR), systemic arterial pressure (SAP), SV, CO, PV and FTc were recorded directly before, during, right after and 5 minutes after RM application (T1 to T4). Kolmogorov-Smirnoff was used to test normal distribution of data and ANOVA was used for the statistical analysis. *P <*0.05 was considered statistically significant.

## Results

HR, SAP, SV, CO, PV and FTc showed a statistically significant decrease during and right after RM compared with the baseline values but they were gradually restored to control values after RM discontinuation (Table 1).

**Table 1:** 

	T1	T2	T3	T3
HR (beats/minute)	69.8±7.1	60.2±10.6*	67.1±7.4*^	68.4±6.9^
SAP mean (mmHg)	89.8±7.7	53.4±5.7*	81.5±7.7*^	87.6±6.7^
SV (ml)	82.2±21.2	22.5±15.5*	75.4±21.7*^	79.6±6.4^
CO (l/minute)	5.75±1.7	1.4±0.9*	5.1±1.7*^	5.5±1.5^
PV (cm/second)	72.6±17.1	26±15.9*	68±16.8*^	70.7±15.9^
FTc (seconds)	0.4±0.06	0.19±0.08*	0.36±0.06*^	0.36±0.06^

*P <0.05 versus T1. ^*P <*0.05 versus T2.

## Conclusion

According to our results, RM application causes a profound impairment of the cardiovascular system. This can be attributed to the increase of the airway pressures, which results in preload decrease and afterload increase of the right ventricle and cardiac contractility attenuation. Nevertheless, these effects are transient and reversible and RM application is a safe technique for the management of intraoperative hypoxemia, provided that adequate cardiac preload is ensured and the anesthesiologist is alert to discontinue the RM if it exceeds cardiovascular reserves.

